# Effects of Different Vitamin D Supplementation Schemes in Post-Menopausal Women: A Monocentric Open-Label Randomized Study

**DOI:** 10.3390/nu13020380

**Published:** 2021-01-26

**Authors:** Addolorata Corrado, Cinzia Rotondo, Daniela Cici, Stefano Berardi, Francesco Paolo Cantatore

**Affiliations:** Rheumatology Clinic, Department of Medical and Surgical Sciences, University of Foggia, 71121 Foggia, Italy; cinzia.rotondo@gmail.com (C.R.); daniela.cici@gmail.com (D.C.); stef.berardi@hotmail.it (S.B.); francescopaolo.cantatore@unifg.it (F.P.C.)

**Keywords:** Vitamin D, calcifediol, cholecalciferol, hypovitaminosis D, muscle strength, Vitamin D supplementation

## Abstract

Background: The improvement of muscular strength is a well-known extra-skeletal effect of Vitamin D. The aim of the study was to evaluate the effectiveness of the calcifediol supplementation compared to various cholecalciferol administration schedules in increasing 25(OH)D serum levels and improving muscular function. Methods: 107 post-menopausal women with hypovitaminosis D were assigned to receive Vitamin D supplementation according to four different regimens: colecalciferol single, monthly, or weekly oral dose and calcifediol weekly oral dose. Serum levels of 25(OH)D and muscular function of lower limbs (Sit-to-Stand test and Timed-Up-and-Go test) were evaluated at baseline and during 6 months follow-up. Results: Calcifediol and weekly cholecalciferol induced a greater and faster increase of serum 25(OH)D, compared to monthly or single-dose cholecalciferol administration. The 25(OH)D increase was associated with an improvement of muscle function of lower limbs. The larger increase of serum 25(OH)D observed with calcifediol and with weekly cholecalciferol was associated with a concomitant greater improvement of muscle strength. Conclusions: Supplementation with calcifediol is more effective and faster compared to cholecalciferol in increasing 25(OH)D serum levels and is associated with a greater improvement of muscular function, thus representing a therapeutic alternative for treatment of hypovitaminosis D.

## 1. Introduction

Vitamin D supplementation is the cornerstone for prevention and management of osteoporosis, as it is associated with a reduction of fracture risk in elderly by contributing to increase bone mineral density (BMD) [1,2]; furthermore, many clinical and experimental studies suggest that the beneficial effects of Vitamin D on fracture risk can be due to the positive effect on muscle strength and the reduction of muscle weakness, which are related to fall prevention [3,4].

Vitamin D receptors (VDR) have been identified in muscle tissue, confirming the role of Vitamin D in muscle health, and the improvement of muscular strength is a well-known extra-skeletal effect of Vitamin D [5]. Hypovitaminosis D is usually asymptomatic, but subjects with low levels of circulating Vitamin D may present proximal muscle weakness, diffuse muscle pain, and difficulty in walking. Nevertheless, there is no consensus on a possible association between circulating levels of Vitamin D and walking speed [6], and only a few studies have assessed the association among serum Vitamin D levels and muscle strength and function in post-menopausal women [7].

The main circulating form of Vitamin D is 25(OH)D, whose half-life is 2–3 weeks and represents the most reliable indicator to monitor the Vitamin D reserves. Conversely, the serum concentrations of the active metabolite 1,25(OH)_2_D are approximately 1000 times lower, and its half-life is nearly 4 h [8,9]. Serum levels of 1,25(OH)_2_Vitamin D do not reflect Vitamin D storage and its measurement is not useful to evaluate Vitamin D status in clinical practice, except in special circumstances [10].

There is no consensus concerning the optimal threshold serum levels of 25(OH)D for bone health and to obtain the different extra-skeletal effects; recommendations vary between 20 and 30 ng/mL, and several studies suggest that the optimal 25(OH)D concentrations should range between 30 and 50 ng/mL to obtain several extra-skeletal effects [11].

The commonest form of Vitamin D supplementation is represented by cholecalciferol (Vitamin D3), and most healthy adults reach the target of 20 ng/mL with 600 to 800 IU Vitamin D per day, whereas the cut off level of 30 ng/mL may require from 1800 IU to 4000 IU vitamin D3 per day [12]. The 25 hydroxylated Vitamin D metabolite (calcifediol) has been suggested as a therapeutic alternative; it has much shorter half-life compared to cholecalciferol and causes a rapid and sustained increase in plasma 25(OH)D concentration [13,14]. The rapidity and the extent of 25(OH)D circulating levels increase depend on the dosage, frequency, and kind of Vitamin D metabolite administration. Several randomized clinical trials showed that calcifediol is more effective and rapid than cholecalciferol in increasing circulating levels of 25(OH) Vitamin D. Furthermore, several reports suggest that different frequencies of supplementation with cholecalciferol act with different potency and rapidity in increasing 25(OH) Vitamin D levels [15,16].

The main aims of the study were: (1) to evaluate the effectiveness of the calcifediol supplementation compared to several cholecalciferol administration schedules in increasing 25(OH) Vitamin D serum levels; (2) to evaluate the effects of calcifediol supplementation compared to several cholecalciferol administration schedule on muscular function in terms of improvement over time of muscular exercise capacity in upper limbs in post-menopausal women. As secondary endpoint, we evaluated the effect of 25(OH)D serum changes on the main parameters of calcium and phosphate metabolism.

## 2. Materials and Methods

### 2.1. Patients and Methods

One-hundred and 60 post-menopausal white females with hypovitaminosis D attending the outpatient clinic of Rheumatology were recruited. Inclusion criteria were serum 25(OH) Vitamin D level ranging from 8 ng/mL to 24 ng/mL, Body Mass Index (BMI) ranging from 18 and 29 kg/m^2^ and good health. Patients taking drugs which could interfere with calcium and phosphate homeostasis or suffering from diseases that can alter the Vitamin D intestinal absorption or that were potentially unable to properly take the Vitamin D supplementation, and unable to perform muscular exercises were excluded. The exclusion criteria were diseases with increased risk of hypercalcemia (sarcoidosis, lymphoma, primary hyperparathyroidism), kidney stones, intestinal malabsorption (celiac disease, lactose intolerance, gastric resection), severe renal impairment, psychiatric disorder, motor disability. Moreover, patients taking antihypertensive drugs, bisphosphonates, anticonvulsant, anticoagulant, corticosteroid, thiazide diuretics, hormone therapy, estrogen receptor modulators for 6 months prior to recruitment were also excluded. Other exclusion criteria were the occurrence of fractures in the last year or fall in the three months before, high intensity exercise, abuse of alcohol, cigarettes, intensive sun exposure in the three months before the study. Demographic and clinical data, including previous Vitamin D and calcium supplementation have been collected at baseline. The enrolled patients were randomly assigned to receive Vitamin D supplementation according to four different regimens, corresponding to an average daily dose of 1000 IU/day: Group 1 = cholecalciferol (D3) 300,000 IU, single oral dose; Group 2 = cholecalciferol 100,000 IU every two months; Group 3 = weekly oral cholecalciferol 7000 IU; Group 4 = weekly oral calcifediol 7000 IU. Patients were randomized using a computerized random number generator. Patients attended the research center every month for blood samples collection and every 20 days for the evaluation of muscular strength.

All patients underwent physical examination and routine blood and urinary analysis at baseline and at each follow-up time point, in a period of time ranging from October until April. Blood samples were collected at baseline and at each follow-up time point. The study was conducted in accordance with the ethics guidelines of the Helsinki Declaration. This study was ancillary to a parallel-group, randomized, placebo-controlled trial which was approved by the medical ethics committee on the 14 May 2014 and accepted by the Italian drug agency. All subjects provided written informed consent before recruitment.

### 2.2. Measurement of Clinical and Metabolic Parameters

Measurement of the biologic serum variables were made on blood samples collected after an overnight fasting of 12 h at baseline, and every month until the end of follow-up (6 months); sera were frozen at −20 °C until assay. The following parameter were evaluated: serum levels of 25(OH)D (chemiluminescent immunoassay kit LIAISON—Diasorin), serum levels of parathormone (PTH) colorimetric immune-enzymatic method) calcium and phosphates (colorimetric method). It is important to note that although chromatographic assay is considered the gold standard for the measurement of 25(OH)D, the automated immunoassays with <10% coefficient of variation (CV) bias can be used in clinical practice. Diasorin Liaison immunoassay kit, which is one of the most frequently used method for research purposes, has a CV <10% and in recent years, it has been reformulated and is now accredited by the Vitamin D Standardization Program (VDSP) as certified to the National Institute of Standard and Technology (NIST) [17].

For each treatment group, at baseline and every 20 days, muscular function of lower extremity was evaluates using the 30 s Sit-to-Stand Test (SST) and the Timed-Up-and-Go (TUG) test. To perform SST, participants were trained to stand from a standard chair and sit back down as many times as possible within a time frame of 30 s, keeping their arms crossed on their chest during testing. The same chair was used for all participants. Participants were encouraged to continue to sit and stand throughout the test. The number of SST repetitions was recorded and represented the unit for this measure. TUG test measures the time (in seconds) taken by a subject to stand up from a chair, walk 3 m (10 feet), turn, walk back to the chair, and sit down [18]. To perform the test, the recruited subjects were instructed to sit on a standard chair, placing his/her back against the back rest, placing his/her arms on armrests, and were invited to rise from a chair, walk on the floor 3 m away, turn around, return, and sit back on a chair. All subjects were allowed to wear their regular footwear. Time measurement began when participants got up from the chair and stopped after they sat back on the chair.

Prior to each test, clear and simple instructions were given orally and were followed by a standardized demonstration. Each participant was allowed one opportunity to practice trial before the actual measurement for both functional tests. The clinician who assessed muscular function tests was blinded to different supplementation regimens.

### 2.3. Statistical Analysis

The results were expressed as mean ± standard deviation (SD) or median and inter-quartile range, when normality is not verified. Qualitative variables are expressed as number and frequency. The time is expressed in months or days. The normal distribution was assessed using the Shapiro–Wilk test. Changes observed at baseline and at the different time points of follow-up in each treatment group were assessed using paired repeated-measures analysis of variance (ANOVA) or Friedman’s test as appropriate Comparisons between groups were assessed using ANOVA followed by Bonferroni test or Kruskal-Wallis test followed by Dunn-Bonferroni test, as appropriate. *p*-value ≤ 0.05 was considered statistically significant. Statistical analyses were performed using IBM SPSS Statistics 23.

## 3. Results

### 3.1. Demographic Characteristics

**Figure 1:**
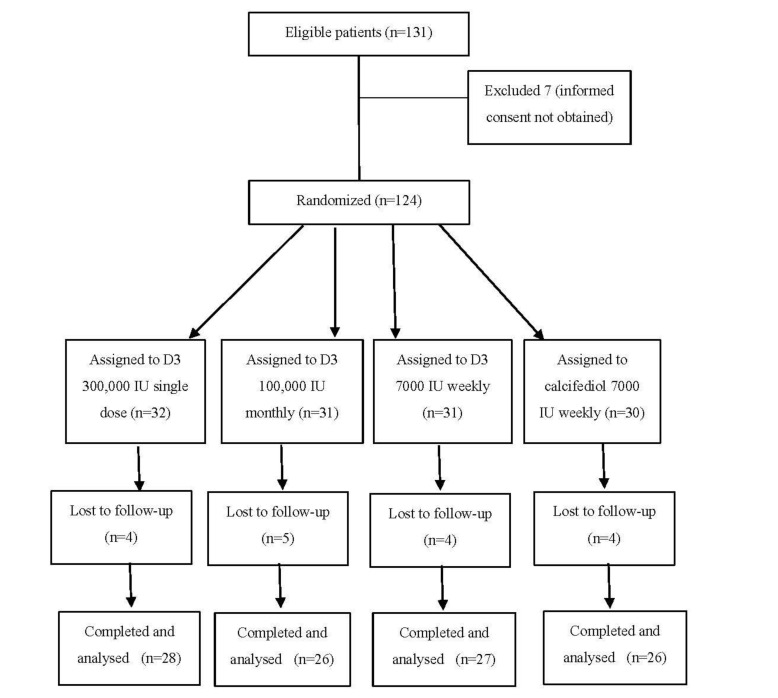
Flowchart of study population.

Overall, 107 subjects completed the study (28 subjects assigned to Group 1, 26 subjects assigned to Group 2, 27 subjects assigned to Group 3, 26 patients assigned to Group 4, Figure 1). All recruited patients were from Apulia, in southern Italy.

At baseline, patients randomly assigned to receive the different Vitamin D supplementation schemes with cholecalciferol and calcifediol showed no differences with respect to BMI, age, sex, and serum levels of 25(OH)D_3_, 1,25(OH)D_3_, calcium, phosphorus. Patients of Group 2 showed a greater serum level of PTH compared to other treatment groups (*p* < 0.05); patients of Groups 3 and 4 showed higher serum levels of alkaline phosphatase (ALP) compared to Groups 1 and 2 (*p* < 0.05). In all groups, both PTH and ALP levels were within normal limits. No differences in tests for measurement of lower extremity muscular function were observed at baseline between the different treatment groups. Data are shown in Table 1.

### 3.2. Effects of Vitamin D Supplementation on Serum 25(OH)D Levels and Serum 1,25-Dihydroxyvitamin D Levels and Biochemical Markers

All Vitamin D supplementation schemes were able to induce an increase of 25(OH)D levels (*p* < 0.0001). Values of 25(OH)D above the threshold of 30 ng/mL were reached at 2 months in patients receiving 7000 IU calcifediol weekly, at 3 months in patients receiving 7000 IU D3 weekly, at 5 months in patients receiving 100,000 IU D3 every two months (nevertheless it should be noted that the mean serum levels of 25(OH)D were just slightly below 30 ng/mL–28.58 ng/mL after 4 months of supplementation) and 300,000 IU D3 single dose. The increase in 25(OH) Vitamin D levels was significant from baseline after 1 month in Group 4 and after 2 months in Group 2 and 3, whereas in Group 1 (single 30,000 UI oral dose of D3) a significant increase of 25(OH) Vitamin D levels appeared after 4 months from the beginning of supplementation. 25(OH)D continued to increase in each treatment group until the end of the study. In subjects treated with calcifediol, the circulating levels of 25(OH)D were significantly higher in each follow-up time point compared to other treatment groups (*p* < 0.001), reaching at the end of the study 66.8 ± 3.98 ng/mL versus 33.68 ± 4 ng/mL in Vitamin D3 single dose, 40.8 ± 3.5 ng/mL in Vitamin D3 100,000 IU every two months and 50.9 ± 4.52 ng/mL in Vitamin D3 7000 IU weekly. Furthermore, in all study groups an increase of 25(OH)D levels at the end of follow-up compared to basal time was observed; in particular 174.4% ± 85.4% in Group 1, 204.2% ± 81.2% in Group 2, 318.8% ± 81.1% in Group 3, and 417.2% ± 113.2% in Group 4. Therefore, among subjects treated with D3, those receiving 7000 IU D3 weekly experienced a greater increase of 25(OH)D levels compared to those receiving 100,000 IU monthly (*p* = 0.0001) and 300,000 IU single dose D3) (*p* = 0.0001). Weekly administration of calcifediol (Group 4) was associated with a significantly greater increase of 25(OH)D serum levels at the end of follow-up, compared to the other treatment groups (*p* = 0.0001).

**Figure 2:**
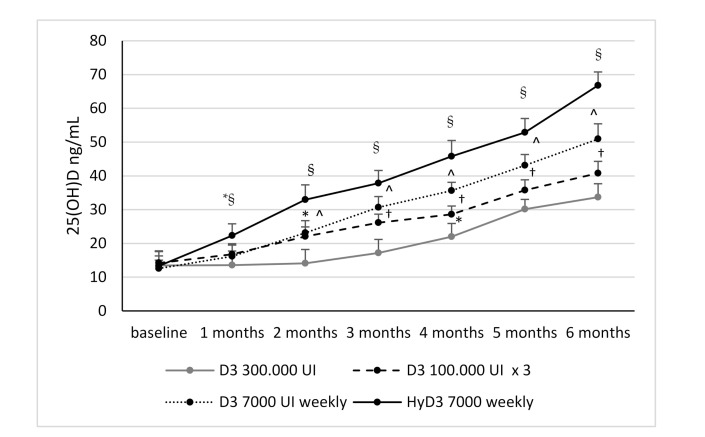
25(OH) Vitamin D serum levels. *: *p* < 0.01 vs. baseline; §: *p* < 0.01 vs. Group 1, 2, 3; ^: *p* < 0.01 vs. Group 1,2; †: *p* < 0.01 vs. Group 1. D3 = cholecalciferol; HyD3 = calcifediol. Group 1 cholecalciferol 300,000 UI single dose; Group 2 cholecalciferol 100,000 every two months; Group 3 cholecalciferol 7000 UI weekly; Group 4 calcifediol 7000 UI weekly.

In patients receiving 300,000 IU single dose D3 a lesser increase in 25(OH)D compared to other treatment groups was observed for the entire follow-up period. No significant differences in increasing of 25(OH)D were found between Group 1 and Group 2 (*p* = 0.233). No significant differences in PTH, calcium and phosphate serum levels were found between supplementation groups during the follow-up period (Table 2). Changes in circulating 25(OH)D levels in all treatment groups during the follow-up time are showed in Figure 2.

### 3.3. Effects of Vitamin D Supplementation on Muscular Lower Extremity Function

**Figure 3:**
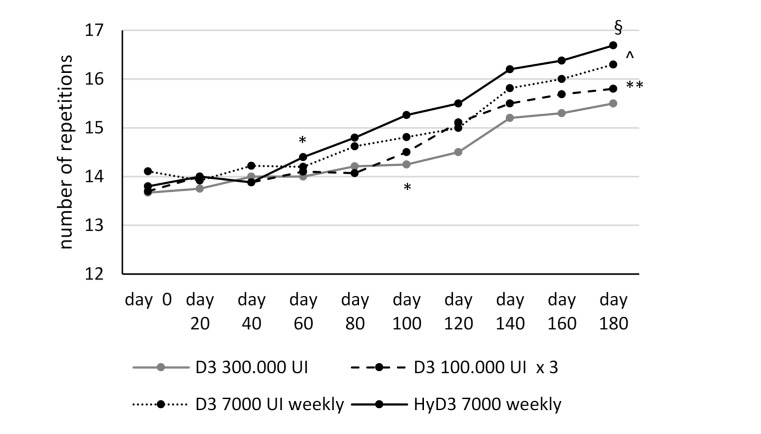
Sit-to-Stand test. *: *p* < 0.05 vs. baseline; §: *p* < 0.05 vs. Group 1, 2, 3; ^: *p* < 0.01 vs. Group 1, 2. **: *p* < 0.01 vs. Group 1. D3 = cholecalciferol; HyD3 = calcifediol. Group 1 cholecalciferol 300,000 UI single dose; Group 2 cholecalciferol 100,000 every two months; Group 3 cholecalciferol 7000 UI weekly; Group 4 calcifediol 7000 UI weekly.

**Figure 4:**
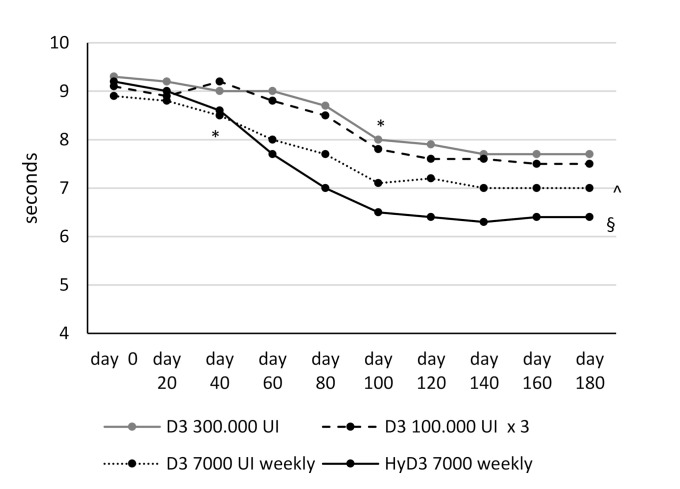
Time Up and Go test. *: *p* < 0.05 vs. baseline; §: *p* < 0.05 vs. Group 1, 2, 3; ^: *p* < 0.01 vs. Group 1, 2. D3 = cholecalciferol; HyD3 = calcifediol. Group 1 cholecalciferol 300,000 UI single dose; Group 2 cholecalciferol 100,000 every two months; Group 3 cholecalciferol 7000 UI weekly; Group 4 calcifediol 7000 UI weekly.

Vitamin D supplementation induced a significant improvement of muscular function evaluated by the functional endpoints TUG test and 30 s repeated SST at the end of the follow-up observation (6 months) with all Vitamin D treatment regimens. The improvement of muscular function from baseline was observed starting from day 60 for repeated SST test and from day 40 for the TUG test, but only in patients treated with calcifediol (both tests) and cholecalciferol 7000 UI weekly (TUG test). In patients treated with cholecalciferol 100,000 UI every two months and 300,000 single dose, the improvement of muscular strength was observed starting from day 100 (both tests). Subjects treated with calcifediol 7000 IU weekly showed a significant greater improvement of lower extremity muscular strength and function evaluated with both tests at the end of follow-up period, compared to patients treated with the different cholecalciferol treatment regimens (*p* < 0.05/*p* < 0.001); furthermore, the positive effects on muscular strength were greater in subject treated with cholecalciferol 7000 IU weekly compared to subject treated with cholecalciferol 100,000 IU monthly or 300,000 IU single dose (*p* < 0.05) ( Figure 3; Figure 4).

## 4. Discussion

The results of this study, according to previously published data [19,20,21] show that Vitamin D supplementation with calcifediol is significantly more potent and more rapid in increasing the serum concentrations of 25(OH) Vitamin D; furthermore, results show that weekly administration of the inactive metabolite cholecalciferol (D3) is associated with a significantly faster and higher increase of 25(OH) Vitamin D serum levels compared to the less frequent once every 2 months or single-dose administration. Concerning the effects of Vitamin D supplementation on muscle function, the results of this study confirm that the improvement of serum levels of 25(OH)D is associated with a concomitant improvement of muscle strength and function of lower limbs, expressed as a reduction of time to perform the TUG test and an increased number of repetitions within 30 s. The greatest and faster increase of 25(OH)D serum levels observed with calcifediol supplementation is associated with a greater improvement of muscle strength; also, the more frequent (weekly) supplementation with cholecalciferol is related to a better muscular outcome, compared to once every two months and single-dose supplementation.

The relationship between different regimens of oral dosing with Vitamin D3, 25(OH)D and 1,25(OH)D is not well established. However, some randomized controlled trial performed on small sample size population showed that the cumulative dose of Vitamin D supplementation may be more important than frequency of dosing [22]. Conversely, according to the presented data a randomized clinical trial including a greater number of patients concluded that dosing frequency significantly affect the 25(OH) serum levels, showing that daily vitamin D3 supplementation is more potent in raising 25(OH)D levels compared to weekly and monthly supplementation while the 25(OH) increase was higher with weekly dose compared to monthly dose [23].

The commonest Vitamin D supplementation is represented by oral cholecalciferol (Vitamin D3). To obtain adequate levels of Vitamin D it has been proposed a daily Vitamin D intake ranging from a 400 IU/day in children aged 0–1 year, to 600 IU/day in children older than 1 year and in adolescents and adults up to 50 years, and 800 IU/day (20 mg) for adults aged over 70 years to maintain the required 25(OH)D concentration [10,24]. Nevertheless, if these amounts of daily intake of Vitamin D are considered enough to ensure an adequate bone health, it is not clear if the same quantities are sufficient to ensure the optimal effects on muscle. Furthermore, to obtain blood levels of 25(OH)D consistently above 30 ng/mL, a daily intake of Vitamin D3 of 1000 UI and 1800 to 4000 UI for subjects up to 20 years and for subjects older than 50 years respectively, may be required [10,13]. Although cholecalciferol represents the most common form of Vitamin D supplementation, other Vitamin D metabolites are available in clinical practice, particularly calcifediol that has much shorter half-life compared to cholecalciferol but is associated with a rapid and sustained increase in plasma 25(OH)D concentration. Several studies comparing cholecalciferol with calcifediol showed that oral calcifediol is more effective and rapid in increasing serum levels of 25(OH)D [25] suggesting calcifediol as a possible therapeutic alternative.

In a randomized, double blind, controlled parallel-group study including post-menopausal women, evaluating multiple dosages of cholecalciferol and calcifediol with different supplementation frequency, calcifediol supplementation given daily, weekly, or as a single bolus was 2–3 times more potent in increasing 25(OH)D3 serum levels than Vitamin D3; plasma 25(OH)D3 concentrations of 30 ng/mL were reached more rapidly and reliably with the 25 hydroxylated Vitamin D metabolite calcifediol [26].

Several open-label studies evaluating different dosages of cholecalciferol and calcifediol confirmed that the efficacy of calcifediol in increasing 25(OH)D levels was significantly higher and faster compared to cholecalciferol, even [20,27,28].

Nevertheless, the efficacy of calcifediol and cholecalciferol supplementation on extra-skeletal effects are controversial, particularly the effects on muscular strength. The crucial role played by 1,25(OH)_2_D in the control of calcium/phosphate homeostasis and in the regulation of bone metabolism and mineralization is well known; insufficient levels of Vitamin D induce rickets in children and osteomalacia in adults [29]. Furthermore, Vitamin D is involved in many physiological processes and exerts a large amount of extra-skeletal effects through different mechanisms [30,31,32,33,34]. VDR is expressed in several cells and tissues, including immune cells, brain, gut, breast, heart and muscle [9,35]. Mice lacking VDR show smaller skeletal muscle fibers and persistence of immature muscle gene expression during adult life [9]. Many clinical and experimental studies confirm a close relationship between Vitamin D and muscle health and support the hypothesis that Vitamin D exerts a positive effect on muscle strength and function.

In subjects with rickets or osteomalacia due to severe Vitamin D deficiency, a characteristic myopathy causing severe weakness of proximal muscle of lower limbs has been described from a long time [36]. Nevertheless, myopathy related to Vitamin D deficit is often underdiagnosed, as the decrease of muscle strength is usually progressive but gradual and a significant loss of muscle strength is necessary before the appearance of impaired muscle function. Therefore, the main initial symptom could be fatigue and only in the late stages of myopathy a more significant muscle weakness, causing inability to walk, could develop [4]. Furthermore, many musculoskeletal symptoms, such as muscle and bone pain, arthralgia, paresthesia are highly unspecific and can be attributed to other rheumatic diseases. Muscular impairment related to Vitamin D deficiency may appear even before the negative bone effect develops and it has been showed that the treatment of myopathy related to hypovitaminosis D requires treatment with a higher dose of Vitamin D [4].

According to the presented study results, a previously published study showed that daily oral supplementation of calcifediol in post-menopausal women aged 50–70 years resulted in higher and faster increase in circulating level of 25 (OH)D compared to Vitamin D3 supplementation, with a concomitant improvement in lower extremity function [21].

Several clinical and experimental studies showed the positive effects of Vitamin D supplementation on muscular strength and the positive correlation between serum 25(OH) Vitamin D levels and lower limbs function [37,38,39,40]. It has been shown that in patients suffering from osteomalacia, Vitamin D supplementation improves muscle strength and reduces the muscle atrophy predominantly observed in type II fibers [40]. Adult subjects with hypovitaminosis D exhibit a significant slower walking speed compared to subjects with normal 25(OH)D status, which can at least in part explain the muscular decline and a greater susceptibility to falls in older subjects [6]. Moreover, supplementation with Vitamin D has proven to have a positive effect in terms of reduction of risk of falls and fractures in elderly [41,42,43]. The effects of Vitamin D on muscle performance have been summarized in several reviews and meta-analysis [6,44,45,46] although some of them reported small and non-significant improvement of muscle exercise capacity. Nevertheless, the available studies evaluating the effect of Vitamin D supplementation on muscle strength and mobility are limited and included small numbers of participants, have been performed on heterogeneous groups, and present large variability in study design, Vitamin D metabolites, type and interval of intervention and outcomes, without non-common protocols, making the real interpretation very difficult; furthermore, the small number of included studies does not permit any firm conclusion [47,48].

As with most of the previously published studies on this topic, the current report is subject to some limitations. One of the main limitations is represented by the single-center design of the study; also, the relatively small number of recruited patients could limit the interpretation of the results, nevertheless it should be considered that most of the available data on the same subject derive from smaller case-series.

Furthermore, it possible to hypothesize that the quickest increase of 25(OH)D induced by calcifediol supplementation could be associated with equally faster 25(OH)D decrease after supplementation discontinuation compared to other regimens, so further studies, performed on a larger number of subjects and a follow-up period of some months after the end of supplementation could be addresses in future research to further validate these preliminary findings.

## 5. Conclusions

Our results show that supplementation with calcifediol is more effective and faster compared to cholecalciferol in increasing 25(OH)D serum levels, without toxicity, so calcifediol can represent a therapeutic alternative. Furthermore, weekly cholecalciferol is more effective and faster compared to single-dose or monthly administration. Increase in circulating levels of 25(OH)D is associated with an improvement of muscular function, very important to prevent falls and fractures in post-menopausal women. Insufficient levels of Vitamin D are partly responsible for proximal muscle weakness of lower limb which in turn is a determinant factor of the appearance of falls, and contributes to increase the risk of fracture. The protective effect of Vitamin D on fractures, in addition to its benefits on calcium homeostasis and bone mineral density, can also be a consequence of the action on muscle strength and muscular function, thus reducing fracture risk through fall prevention.

Overall, the small number of studies and the high degree of heterogeneity precludes any firm conclusions, although further investigations are certainly warranted.

## Figures and Tables

**Table 1:** Baseline characteristics of treatment groups.

	Group 1 (28) D3 300,000 IU Single Dose	Group 2 (26) D3 100,000 IU Monthly	Group 3 (27) D3 7000 IU Weekly	Group 4 (26) HyD3 7000 IU Weekly	*p*
Age	60.51 ± 5.1	58.3 ± 7.4	63.4 ± 5.5	60.9 ± 8.1	ns
BMI	24.8 ± 2.3	25.9 ± 0.45	23.8 ± 1.5	23.3 ± 1.2	ns
SST (*n*)	13.6 ± 1.07	13.7 ± 1.19	14.1 ± 0.8	13.8 ± 1.14	ns
TUG (seconds)	9.36 ± 1	9.19 ± 1.03	8.9 ± 1.01	9.2 ± 1.04	ns
Serum 25(OH)D	13.46 ± 4.3	14.2 ± 3.3	12.5 ± 2.46	13.3 ± 2.9	ns
Serum Ca	9.9 (0.93)	9.9 (0.60)	9.4 (1.8)	9.1 (1.5)	ns
Serum P	3.35 (0.3)	3.3 (0.7)	3.3 (0.28)	3.6 (0.35)	ns
PTH	33.5 (18.28)	37 (6.27)	35.5(4.1)	28.3 (8.08) *	0.0001
ALP	50.5 (12.17)	50.1 (8.45)	73.2 (9.4) **	70.5 (7.3) **	0.0001

TUG = Timed-Up-and-Go; SST = Sit-to-Stand Test; Ca = Calcium; P = phosphates; PTH = parathormone; ALP = Alkaline Phosphatase; D3 = cholecalciferol; HD3 = calcifediol. * *p* < 0.05 Group 2 vs. Groups 1, 3, 4; ** *p* < 0.05 Groups 3, 4 vs. Groups 1, 2. Results for age, BMI, SST, TUG, serum 25(OH) for which normality was verified, are expressed as mean ± SD. Serum Ca and P, PTH, and ALP that are not normally distributed are expressed as medial and inter-quartile range.

**Table 2:** Parameters of calcium and phosphate metabolism.

		Baseline	1 Month	2 Months	3 Months	4 Months	5 Months	6 Months
**Group 1**	Ca	9.9 (0.9)	9.7 (0.8)	9.6 (0.8)	9.7 (0.5)	9.6 (0.6)	9.6 (0.6)	9.4 (0.5)
	P	3.3 (0.3)	3.5 (0.4)	3.5 (0.5)	3.5 (0.5)	3.5 (0.5)	3.6 (0.5)	3.5 (0.4)
	PTH	33.5 (18.3)	30.6 (9.7)	30 (6.3)	33.1 (11.7)	32.1 (8.4)	33.4 (7.8)	32.8 (10.4)
	ALP	50.5 (12.2)	66.8 (12.8)	67 (8.2)	67.3 (5.1)	69.2 (6.4)	69.2 (9.7)	70.3 (10.3)
**Group 2**	Ca	9.9 (0.6)	9.7 (0.5)	9.4 (0.5)	9.4 (0.6)	9.6 (0.6)	9.5 (0.4)	9.5 (0.5)
	P	3.3 (0.7)	3.3 (0.5)	3.6 (0.2)	3.5 (0.3)	3.5 (0.4)	3.6 (0.4)	3.6 (0.6)
	PTH	37 (6.3)	40.1 (7.7)	35.7 (7.5)	39.5 (8.3)	44.4 (7.8)	41.4 (6.3)	42.7 (13.1)
	ALP	50.1 (8.4)	71.4 (11.1)	64.8 (9.2)	69.7 (9.9)	62.2 (8.1)	61.3 (7.6)	66.4 (7.5)
**Group 3**	Ca	9.4 (1.8)	9.4 (0.7)	9.5 (0.7)	9.6 (0.4)	9.4 (0.6)	9.6 (0.7)	9.5 (0.5)
	P	3.3 (0.4)	3.4 (0.5)	3.5 (0.6)	3.5 (0.5)	3.5 (0.5)	3.5 (0.5)	3.5 (0.5)
	PTH	35.5 (4.1)	40.2 (8.3)	40.2 (8.3)	40 (12)	41.3 (7.5)	38.4 (5.8)	34.2 (7.7)
	ALP	73.2 (9.4)	68.9 (13.9)	70.5 (9.4)	71.2 (8.9)	71.9 (6.3)	70.2 (6.6)	70.2 (8.5)
**Group 4**	Ca	9.1 (1.5)	9.4 (0.7)	9.1 (0.8)	9.2 (0.5)	9.1 (0.7)	9.4 (0.5)	9.3 (0.6)
	P	3.6 (0.4)	3.6 (0.5)	3.6 (0.6)	3.7 (0.8)	3.7 (0.7)	3.6 (0.7)	3.7 (0.9)
	PTH	28.3 (8.1)	30.2 (7.3)	30.1 (9)	30 (6.2)	32.9 (7.1)	33.5 (8.2)	31.2 (6.7)
	ALP	70.5 (7.3)	75.9 (10.3)	69.5 (6.1)	70.1 (9.1)	70.1 (4.8)	70.3 (12.4)	69.5 (12.5)

Ca = Calcium; P = phosphates; PTH = parathormone; ALP = Alkaline Phosphatase. Results are expressed as median and inter-quartile changes.
